# Special Issue: Fungal Endophytes in Plants

**DOI:** 10.3390/jof4030104

**Published:** 2018-09-01

**Authors:** Gary Strobel

**Affiliations:** Montana State University, Dept of Plant Sciences, Bozeman, MT 59717, USA; uplgs@montana.edu

Once in a while scientific developments occur that represents a complete shift in the paradigm of a scientific discipline. The emerging field of endophyte biology is an example of this phenomenon. One hundred years ago the search was on to describe the pathology and causal agent of every plant disease on the planet. Every microbe associated with a plant was suspect. Those not having a pathological etiology were discarded as uninteresting and not worthy of attention. By the mid-twentieth century some investigators began to systematically isolate, describe and name the non- pathogens of plants and they were designated as endophytes if they were found in the living tissues of the plants. Soon it was learned that some of these organisms associated with certain grasses were the cause of abortions and death in livestock. The name endophyte took on a whole new meaning as an unwanted biological case. With the advent of the discovery of certain endophytes making the drug-taxol there was another shift in the thinking and potential importance of these microorganisms. The field of endophyte biology took on a whole new persona. Soon it was realized that there are literally thousands of endophytes making hundreds of compounds that may have uses in industry, medicine and agriculture. Further studies have revealed that plants have a microbiome just as humans and other animal species and that this assemblage of microbes has an important role in the ultimate health and survival of plant species. We are just now putting resources and time into the study and ultimate utilization of these microbes to the benefit of mankind. Some endophytes seem to provide protection to the plant from other microbes, insects and herbivores, others promote growth while others are known that allow the plant to withstand environmental stresses such as heat and drought. The microbiome of plants mainly represents fungi but bacterial species also occur with some regularity.

My career began as a student of forestry at Colorado State University in the mid-1950s. I soon learned that this discipline was not organized to address the fundamental questions of science so I became a botany major with a minor in chemistry. Eventually, I went on the UC-Davis in California for a PhD in plant pathology. Again, I felt that chemistry, biological chemistry and microbiology held the answers to the future of plant biology. I became an assistant professor of plant pathology at Montana State University in 1963. I focused on questions relating to the fundamental aspects of plant disease physiology and biochemistry. Phytotoxins, plant receptors and other major topics concerning the biochemistry of plant disease were the targets of my time and energies. In the early 90s we successfully isolated and characterized taxol from a novel endophytic fungus associated with a pacific yew tree growing in Northern Montana. Soon we began to examine endophytes from forests all over the planet. Those trips took us to some of the most beautiful, enchanting, and challenging places and those experiences have left an indelible mark on my memory. By now some of these discoveries have found their way into the real world such as the use of *Muscodor albus* as a soil treatment as a replacement for the use of methyl bromide. This endophytic organism has been approved by the US-EPA and is now on the market. Furthermore, the volatile antimicrobial compounds of other Muscodor isolates will soon appear in the marketplace.

It has been an honor to me to have been asked to serve as the editor of this special volume on endophytes. It caps a long career dedicated to an understanding of how microbes associate with plants and how we can utilize this information to improve crop production as well as our own lives. Certainly, the efforts of the *Journal of Fungi* staff including managing editor Aimar Xiong, assistant editors Ashlynn Wang and Monica Cui are acknowledged as being helpful while being at the same time being professional and courteous. As someone once said—“If I had to do this career all over again- I would.” The strange and interesting world of fungi offers to us a never-ending challenge of excitement and discovery.

